# Inhaled Anesthetics Promote Albumin Dimerization through Reciprocal Exchange of Subdomains

**DOI:** 10.1155/2010/516704

**Published:** 2010-03-24

**Authors:** Benjamin J. Pieters, Eugene E. Fibuch, Joshua D. Eklund, Norbert W. Seidler

**Affiliations:** ^1^Department of Anesthesiology, University of Missouri-Kansas City School of Medicine, 4401 Wornall Road, Kansas City, MO 64111, USA; ^2^Biochemistry Department, Kansas City University of Medicine and Biosciences, 1750 Independence Avenue, Kansas City, MO 64106, USA

## Abstract

Inhaled anesthetics affect protein-protein interaction, but the mechanisms underlying these effects are still poorly understood. We examined the impact of sevoflurane and isoflurane on the dimerization of human serum albumin (HSA), a protein with anesthetic binding sites that are well characterized. Intrinsic fluorescence emission was analyzed for spectral shifting and self-quenching, and control first derivatives (spectral responses to changes in HSA concentration) were compared against those obtained from samples treated with sevoflurane or isoflurane. Sevoflurane increased dimer-dependent self-quenching and both decreased oligomer-dependent spectral shifting, suggesting that inhaled anesthetics promoted HSA dimerization. Size exclusion chromatography and polarization data were consistent with these observations. The data support the proposed model of a reciprocal exchange of subdomains to form an HSA dimer. The open-ended exchange of subdomains, which we propose occuring in HSA oligomers, was inhibited by sevoflurane and isoflurane.

## 1. Introduction

Inhaled anesthetics affect protein-protein interaction by disrupting heteromeric [1] and homomeric [2] binding in some cases and by promoting such interactions in others [3, 4]. These observations warrant further examination of the interfacial cavities that regulate protein-protein interaction. Human serum albumin (HSA) provides a useful model in studying these processes, since it can form dimers [5] and oligomers [6] in a concentration-dependent manner [7]. HSA has three homologous domains (I–III), each with two subdomains (a, b), and inhaled anesthetics bind at several sites [8–13], one of which is located at the cleft between subdomains IIa and IIb. This high-affinity site is at the external side of a cavity that contains the protein's only tryptophan (W214), whose properties were tracked fluorometrically. We recently proposed [14] that this site can interchange with a site on another HSA forming reciprocal intersubdomain interactions in a dimeric conformation. The current study suggests that this process is modulated by inhaled anesthetics.

## 2. Materials and Methods

### 2.1. Preparation of Samples

HSA (10 mg/mL; Sigma-Aldrich: A-1653, A-8763) was dissolved in a 10 mM sodium phosphate buffer (pH = 7.4), passed through a 0.2 *μ*m filter, diluted to 0.5–6.0 mg/mL and allowed to equilibrate. HSA was approximately 99% pure as determined by agarose gel electrophoresis and was essentially free of globulins. Samples (1.0 mL) were treated with and without sevoflurane (12 *μ*L; 15 minutes on rotator at 22°C) and then assayed as described below. Sevoflurane (Abbott; bp: 58.6°C; density: 1.52 g/cm^3^) and isoflurane (Hospira; bp: 48.5°C; density: 1.50 g/cm^3^) were layered with argon following use. In order to effectively assess the role of agent on HSA self-association at various protein concentrations, it was necessary for us to use saturating levels of sevoflurane and isoflurane (12 *μ*L in 1.0 mL samples), which are immiscible in water and appear as a very small bolus at the base of the test vessel. Thus, all of the binding sites that were previously identified in the literature would be likely saturated. By attempting to use substantially lower volumes, a bolus would not form resulting in a coat on the surface that volatilizes rapidly, leaving an uncertainty associated with partial binding of agent particularly since protein concentration was varied. Under the conditions used in this study, protein denaturation does not occur [15], and HSA returned to its native state approximately 21 hours after removal of agent (data not shown).

### 2.2. Fluorescence Spectroscopy

**Figure 1:**
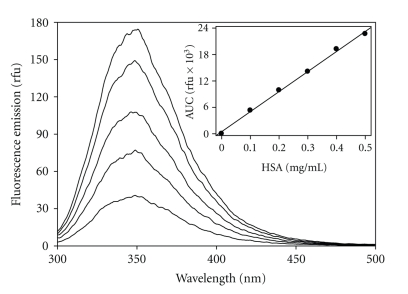
Fluorescence spectra at low HSA concentrations. Tryptophan fluorescence spectra were obtained as described in Materials and Methods. Ascending tracings represent HSA at 0.1, 0.2, 0.3, 0.4, and 0.5 mg/mL, respectively. Inset, plot of the AUC (areas under the curve of entire spectral region) against HSA concentration (*r* ^2^ = 0.997).

Tryptophan emission spectra (e.g., 293 nm; em, 300–500 nm; 2.5 nm slits; 600 nm/min; 290 nm em cutoff) were obtained at various HSA concentrations using a Perkin Elmer LS50B luminescence spectrometer. Values for the center of spectral mass (CSM = *∑*(*ν*F*i*)d*i*/*∑*F*i *d*i*, *i *= 33.3 cm^−1^), where (*ν*) is wavenumber and (F*i*) is emission intensity, were calculated from the emission spectra. They indicate spectral position and reflect the conformational events that change hydration at the W214 [16]. We calculated a parameter from the spectral data that reflects the magnitude of concentration-dependent self-quenching [14]. Fluorescence intensities of HSA at concentrations below 0.5 mg/mL were measured as areas under the curve (Fo = *∑*F*i *d*i*), showing a linear increase with protein concentration (Figure 1) and demonstrating that there was no self-quenching.

**Figure 2:**
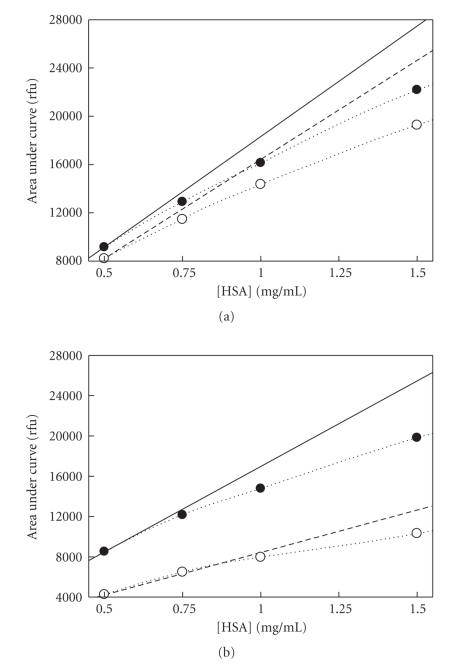
Observed fluorescence shown relative to projected unquenched maxima. Extrapolation of regression lines were drawn from fluorescence data that were obtained at low HSA concentrations from control (solid line), sevoflurane-[A] and isoflurane [B]-treated (dashed line) samples. The projected unquenched maxima are compared with data (dotted line) observed at the concentrations shown (control, closed circles; sevoflurane-[A] and isoflurane [B]-treated, open circles). The difference between the regression lines and the dotted lines represents IQU.

Regression lines (Figure 2) were extended to give a projected Fo for control and for samples treated with agent. At higher HSA concentrations, fluorescence (F) intensities deviated from the linear trajectory (Figure 2) similar to the self-quenching that occurs in the homomeric assembly of protein ionophores [17]. The difference between the extrapolated linear projection (Fo) and the observed F (IQU = Fo − F) provides a useful parameter, which we termed integrated quench units (IQUs), that represents the magnitude of quenching at each of the protein concentrations tested.

Solute quenching studies, which use the Stern-Volmer expression, Fo/F = 1 + *kQ*, where *Q* is solute concentration, assume a bimolecular process [18]. This approach involves fixing protein concentration, which acts as the fluorophore, and varies quencher concentration. In our study, we varied the fluorophore concentration, or HSA, which acts also as the quencher, making the term, Fo/F, inappropriate. The difference term, Fo − F, is commonly used with fluorophores that self-quench [19] and with differentially quenched fluorophores in a single protein [18]. The quenched complex (dimer/oligomer) is implied by the expression, Fo − F = IQU, which was tracked as a function of HSA concentration and dependent upon at least two equilibria (monomer/dimer and monomer/trimer). IQU and CMS were calculated from the spectra obtained at various concentrations of HSA in the presence and absence of sevoflurane or isoflurane. From these discrete data, first derivatives and second derivatives were calculated as previously described [14]. The parameters used in this study (i.e., first derivatives) did not lend themselves well for investigation of dose-response relationships. Tryptophan polarization was also measured at ex/em of 293 nm/345 nm (5 nm slits; 10 s integrations) and data given in millipolarization (mP) units.

### 2.3. Size Exclusion Chromatography

Samples were run through a gel filtration column (1.5 × 12 cm; agarose-based BioGel A 1.5 m fine) using a BioLogic LC system (BioRad). A sodium citrate (10 mM, pH = 6.4) elution buffer at a 1.0 mL/min flow rate was used with UV detection (280 nm absorbance). Quantitation of monomers and dimers were performed by either integration of absorbances (Sigma Plot 11.0) or peak height measurements. Comparisons to assess the effects of sevoflurane involved *t*-tests or one-way ANOVAs with Bonferroni posttests. Calibration standards gamma globulin (158 kDa) and ovalbumin (44 kDa) were obtained from BioRad.

## 3. Results

**Figure 3:**
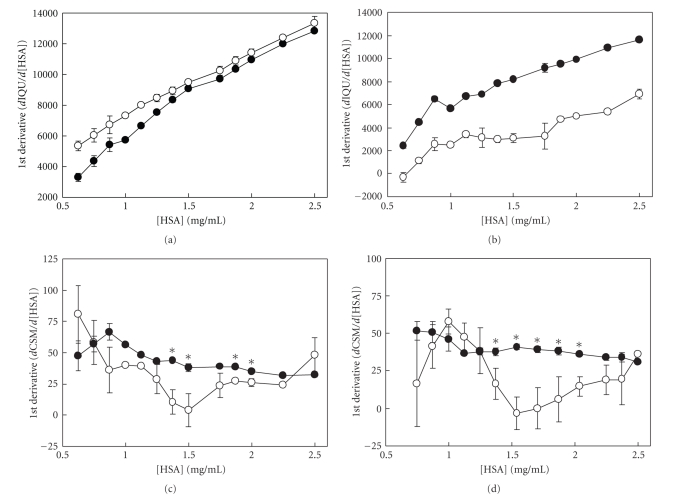
Effects of sevoflurane and isoflurane on the first derivatives of CSM and IQU. HSA was treated with (open circles) and without (filled circles) sevoflurane (a) and (c) and isoflurane (b) and (d) prior to measuring fluorescence spectra and determining the first derivatives of IQU (a) and (b) and CSM (c) and (d). Data were from four independent experiments and presented as M ± SEM. Asterisks indicate differences (*P* < .05) due to anesthetic agent.

In order to assess the effects of anesthetic agent on HSA dimerization, we examined the spectral response to changes in HSA concentration. The HSA concentration range at which dimers and oligomers form was previously established [7, 20]; as HSA increases, the amount of oligomers increases as well as the size of the multimer from trimer to tetra-, penta-, and hexamers. Once the trimer forms, it is likely the multimerization event is monomeric addition to a polymer. This event can be examined at lower concentrations which allow for fluorometric analysis without interference due to higher concentrations. The purpose of this study was to examine the conformational transitions that are initially expressed at the concentration range used, which may provide insight into the behavior of target CNS proteins that exist in the low micromolar range. In untreated samples, IQU and CSM increase with HSA concentration exhibiting a biphasic response [14], suggesting that self-association affects the W214 and that there are different conformational events for dimerization and for postdimer oligomerization. With sevoflurane, first derivatives of IQU were greater than control during HSA dimerization over the 0.6 to 1.3 mg HSA/mL range (Figure 3(a)), suggesting that sevoflurane promoted protein-protein interactions that enhanced self-quenching. 

With isoflurane, first derivatives of IQU were decreased at all concentrations examined (Figure 3(b)). These disparate observations may be attributed to differential occupancy at the subdomain IIa binding site. We previously showed that second derivatives of IQU from control samples below 1 mg HSA/mL differed reproducibly from that obtained at concentrations above 1 mg HSA/mL [14]. Interestingly, a comparison of second derivatives of IQU between control and treated samples exhibited differences only in the postdimeric range from 1.0 to 1.75 mg HSA/mL for both agents (control: 4723 ± 465.6 versus isoflurane: 663 ± 582.3, M ± SEM, *P* <. 0005 and control: 5772 ± 359.5 versus sevoflurane: 3957 ± 161.8, *P* < .001). All other comparisons of control versus treated samples were found to be not significant, suggesting that anesthetic agent exhibited the greatest effect in the range thought to involve the early forms of oligomers beginning with trimers [7]. These results were consistent with those involving CSM. With sevoflurane, first derivatives of CSM were less than control in the postdimeric or early oligomeric phase over the 1.4 to 2.0 mg HSA/mL range (Figure 3(c)), suggesting a stabilization of HSA dimers. With isoflurane, first derivatives of CSM were also less than control in precisely the same postdimeric range from 1.4 to 2.0 mg HSA/mL (Figure 3(d)).

**Figure 4:**
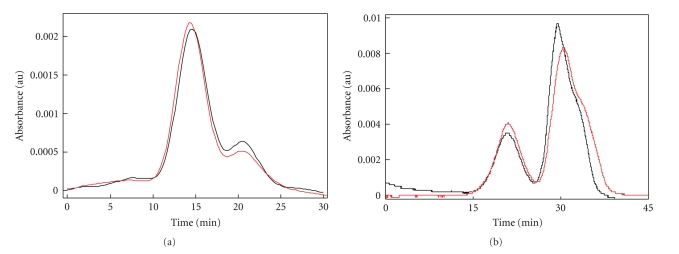
Anesthetic agents promote albumin dimerization. HSA[A] and BSA[B] were treated with (red) and without (black) agent (sevoflurane [A] and isoflurane [B]) prior to size exclusion chromatography and quantitation of the peaks. Tracings in (a) are smoothed curves that represent averages from four independent experiments. Tracings in (b) are from a representative experiment. Column size and flow rates differed slightly between (a) and (b) with gamma globulin (158 kDa) eluting at 13.1 and 19.2 minutes, respectively.

Gel filtration chromatography of sevoflurane-treated samples (1.0 mg HSA/mL) exhibited a different distribution of monomers and dimers compared with control (Figure 4(a)). Sevoflurane treatment promoted dimer formation (*P* < .05). Additionally, the dimers from sevoflurane-treated samples eluted earlier than those of control (*P* < .04). The relative amount of monomers was less with sevoflurane (*P *< .03), suggesting a change in the monomer-dimer equilibrium. Isoflurane showed a similar effect (Figure 4(b)) at 0.675 mg BSA/mL with the predicted shift in protein distribution to favor the dimer in the presence of isoflurane. At these concentrations there were no detectable differences in the amount of higher-order oligomers. 

**Figure 5:**
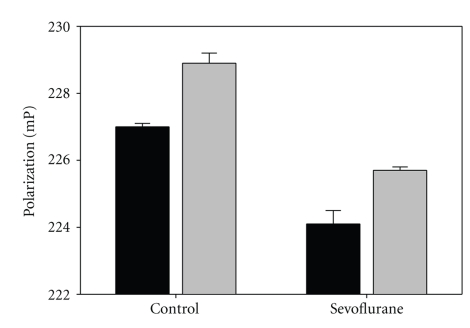
Sevoflurane decreased intrinsic fluorescence polarization. HSA (10 mg/mL) was treated with sevoflurane and then analyzed for fluorescence polarization and compared against control with (gray bars) and without (black bars) isotonic saline. Data are from multiple readings from two independent experiments and presented as M ± SEM.

When HSA (10 mg/mL) was treated with sevoflurane and then analyzed for tryptophan fluorescence polarization, we observed a decrease relative to control (Figure 5) in either low (control: 227.0 mP; plus sevoflurane, 224.1, *P* < .02) or high ionic strength (control: 228.9; plus sevoflurane: 225.7, *P* < .005), consistent with a destabilization of higher-order oligomers in favor of the smaller more mobile dimer.

## 4. Discussion

The three homologous domains of HSA exist in a V-shape. The apposing lobes represent domains I and III. The vertex represents the interface of subdomains IIa and IIb, which has a cluster of hydrophobic residues, including V216, V231 (helix 2 and 3, subdomain IIa) and L331, L347 (helix 2 and 3, subdomain IIb), and has a salt bridge between residue R209 (helix 2, subdomain IIa) and residues D324 and E354 (helix 2 and 3, subdomain IIb). We previously proposed [14] that dimerization results from a reciprocal intersubdomain switch between two HSA molecules, such that subdomains IIa and IIb of one HSA interact with the complementary subdomains (IIb and IIa) of another HSA. The model further suggests that higher-order oligomers emerge from sequential intersubdomain exchanges from a unique trimeric structure that has free subdomains capable of extension. It is fortuitous that HSA has a single tryptophan residue (W214) that is on helix 2 in subdomain IIa. Although W214 is oriented internal to subdomain IIa, the fluorometric properties are affected by changes in protein concentration in predictable ways that cause self-quenching and spectral shifts [14], suggesting that the vertex plays a role in protein-protein interaction. Others have also suggested that W214 is at the interface of the HSA dimer [21].

Interestingly, halothane binds [10] at the site represented by the vertex. Three halothane molecules bind to this site and are within 5 angstrom of the residues described above (1E7C, pdb). Inhaled anesthetics therefore may modulate events such as the proposed mechanism of HSA dimerization and oligomer formation. We examined whether anesthetic agent affected protein-protein interaction by comparing first derivatives of IQU, which measures self-quenching, and CSM, which measures fluorophore hydration, over a range of HSA concentrations in the presence and absence of agent. The differences that we observed suggest that the proposed IIa/IIb intersubdomain switch is influenced by inhaled anesthetics.

Since spectral changes were compared as first derivatives, meaning that they were a function of changes in protein concentration in the range that dimerization and early oligomers form, the simplest interpretation would be that homomeric associations contributed to these findings. Any other interpretation would be inappropriate. Our speculation is limited to the mechanisms of subunit interaction that may contribute to the changes. And since HSA contains only one single tryptophan, its microenvironment is implicated in the proposed mechanism. Sevoflurane increased the IQU response (Figure 3(a)) over a range of HSA concentrations (0.68–1.25 mg/mL) that is associated with dimer formation [7, 20, 22]. It was previously shown that self-quenching of intrinsic fluorescence occurs upon dimerization of *β*-lactoglobulin [23]. The self-quenching indicates a close proximity of native fluorophores (W214) on neighboring HSA molecules during dimerization. This observation suggests that sevoflurane increased the concentration-dependent self-quenching by promoting HSA dimerization in a manner consistent with the proposed mechanism. With isoflurane, first derivatives of IQU were decreased at all concentrations examined (Figure 3(b)), likely a feature of the greater quenching effects of isoflurane on monomeric HSA (Figure 2) that occur independent of changes in HSA concentration. Nevertheless, in examining second derivatives of the IQU parameter, both sevoflurane and isoflurane exhibited similar effects at the postdimeric concentrations of HSA, suggestive of a stabilization of the HSA dimeric structure or a destabilization of early oligomers. This observation is supported by size exclusion chromatography of HSA samples (1.0 mg/mL) treated with sevoflurane (Figure 4(a)) that demonstrated increased dimer and decreased monomer formation. The decrease in elution time of the dimer may be due to added mass, which is consistent with the previously documented eight molecules of anesthetic agent bound per HSA subunit [10], contributing about 3.2 kD to the dimer. Alternately, the dimer conformation in the presence of agent may exhibit a larger surface area, which is indicative of expansion and consistent with the previous observations [12].

Sevoflurane and isoflurane also decreased the CSM response (Figures 3(c) and 3(d)) over the range of HSA concentrations (1.4 to 2.0 mg/mL), where trimers begin to appear [7]. This agent-induced decrease in CSM response occurred in the post *K*d (HSA monomer-dimer) concentration range. The CSM response, which is the concentration-dependent blue-shift of the W214 emission spectrum, indicates a loss of hydration [16] in this region due to the protein-protein interaction in oligomerization [24, 25]. Our observation suggests that sevoflurane and isoflurane brings about a stabilization of the dimer, a destabilization of the early trimers, or an inhibition of metastable oligomers. Support for this suggestion is in the observation that sevoflurane decreased the fluorescence polarization of HSA samples (Figure 5). The literature provides evidence that self-quenching of fluorescein-labeled HSA is accompanied by self-depolarization [26], indicating homotransfer.

Protein-protein interaction is a process that normally regulates the function of many neuronal membrane proteins [27] and may be the mechanism by which inhaled anesthetics alter the biological activity of target proteins. Some of the effects that we examined with HSA may be generalized to CNS receptor targets, such as the GABA_A_ receptor [28]. It would be of interest to determine if the GABA_A_ receptor also undergoes reciprocal subdomain exchange. Dimerization involves interfacial cavities whose dimensions and properties are dependent on the packing density of protein side chains [29]. Sevoflurane or isoflurane may alter the geometry of these cavities, stabilizing the quaternary structure of proteins in a unique manner. Interestingly, parvalbumin reaches a monomer-dimer or monomer-trimer equilibrium depending on presence of divalent cations [30]. Furthermore, static quenching of W214 by nearby residues may occur [31], suggesting a conformational realignment from a reciprocal exchange or swap (i.e., dimer) to an open-ended exchange of subdomains (i.e., trimer as a nucleation center for oligomerization).

Of the multiple sites to which anesthetic agent bind, the drug binding sites I and II at subdomain IIa and IIIa, respectively, and the intersubdomain cleft between subdomains IIa and IIb are the sites most studied in the literature. The intersubdomain cleft interfacing subdomains IIa and IIb is likely the site to which the agents examined in the present study had their effect, as this site suggests a swapping of subdomains that would have an impact on the tryptophan signal. The concentration-independent effects of isoflurane differed from sevoflurane and their IQU first derivatives also differed, suggesting that these two agents likely show different affinities to the internal cavity of the subdomain IIa, which would directly quench the tryptophan signal. Nevertheless, the corroboration of the CSM data and the second derivatives of IQU support the working model that upon binding to the interface at subdomains IIa and IIb dimerization appears favored over the early oligomeric structures.

 HSA dimerizes more readily without fatty acids [5], and inhaled anesthetics displace fatty acids from this intersubdomain cleft [10], suggesting that the formation of these interfacial cavities may be due to acyl chain displacement. This mechanism may be applicable to neuronal membrane proteins. 

Isoflurane, which binds to drug binding site II in subdomain IIIa at the Y411 residue [32], also binds to the interdomain cleft and likely drug binding site I in subdomain IIa affecting tryptophan signal [11]. We observed a large quenching of the monomeric HSA (Figure 2) consistent with [11]. Sevoflurane and isoflurane showed identical effects on CSM suggesting similar binding profiles (Figure 3). Sevoflurane also affected W214 fluorescence in a manner that was independent of changes in protein concentration. Monomeric HSA (0.5 mg/mL) in the presence of sevoflurane exhibited a 13.8% decrease in emission intensity and a blue-shifted spectrum (lambda-max: 351.3 nm, control; 349.2 nm, plus sevoflurane). These direct affects indicated that sevoflurane did also bind to drug-binding site I, which is an internal cavity of subdomain IIa. This observation is consistent with the binding of isoflurane and halothane in HSA [11], though isoflurane had a greater quenching effect on monomeric HSA (Figure 2). In our study, these measurements were used to normalize the data that assessed the effects of protein concentration.

We observed that inhaled anesthetics had concentration-dependent effects on HSA in that dimerization was promoted, as evidenced by first and second derivatives of IQU and that postdimer oligomerization was inhibited as evidenced by a decrease in first derivatives of CSM at 1.4 mg HSA/mL. The data support a dimer model that exhibits a reciprocal intersubdomain conformation that must rearrange to generate oligomers. Reciprocal exchange of subdomains was previously identified as a mechanism of homo- and heterodimerization of transcriptional regulators containing helix-loop-helix domains [33], which show similarities to the behavior of helix 2 and 3 of HSA subdomains IIa and IIb, and may be a general mechanism for protein assembly [34].
